# A call for inclusive public health communication to promote the health of neurodivergent communities during and post-COVID-19

**DOI:** 10.1016/j.puhip.2023.100388

**Published:** 2023-05-05

**Authors:** Emily Hotez, Shanice Hudson, Asal Bastani, Laila Khorasani, Holly C. Hohmeister

**Affiliations:** a University of California, Los Angeles (UCLA), David Geffen School of Medicine, Department of General Internal Medicine / Health Services Research, 911 Broxton Ave, Los Angeles, CA, 90095, USA; b The Hood Medicine Initiative, USA; c The Association of University Centers on Disability (AUCD), 1100 Wayne Ave., Suite 1000Silver Spring, MD 20910, USA

## 1 Overview

Individuals with intellectual and developmental disabilities (i.e., neurodivergent [ND] individuals) experienced disproportionate health and collateral impacts from the COVID-19 pandemic [1,2]. Inclusive public health communication—defined as health-related messaging that includes and prioritizes ND populations in public health preparedness and response efforts [3]—represents an opportunity to ameliorate these and future health inequities. As interdisciplinary researchers, practitioners, and individuals with lived experience related to ND and intersectional populations, the overarching goal of this commentary is to promote uptake of inclusive public health communication across systems and sectors. With this goal in mind, we focus this commentary on three key topics pertaining to inclusive public health communication that are increasingly cited in the evidence base: 1) intersectionality and healthcare bias; 2) social media utilization; and 3) strategies for establishing trust with ND populations. In doing so, we endeavor to spur research on inclusive public health communication, as well as bolster the inclusive public health capacities of diverse ND stakeholders.

## 2 Background

In the U.S., 1 in 6 is neurodivergent (ND)—that is, has an intellectual and/or developmental disability (I/DD), including ADHD, autism, cerebral palsy, or developmental delays, with or without intellectual impairment, as well as hearing, vision, and speech impairments [4]. Despite the proliferating neurodiversity movement that seeks to: 1) respond to ND individuals’ diverse needs, experiences, and preferences; and 2) promote their health, well-being, and thriving—rather than find a “cure” or “normalize” [5]—ND individuals continue to experience significant negative health outcomes across the life course. Relative to the general population, ND individuals are at increased risk of cardiovascular disease, diabetes, epilepsy, psychiatric conditions, emotional difficulties, gastrointestinal disorders, mobility challenges, and other chronic conditions, as well as lower overall health and life expectancy [6].

Negative health outcomes experienced by the ND population are partially due to social determinants of health (SDOH): modifiable social and environmental conditions that impact a wide range of health, functioning, and quality-of-life outcomes and risks [7]. These include lifelong stigma, discrimination—particularly ableism—and marginalization across interpersonal and healthcare contexts; systemic barriers to engaging in health-promoting behaviors; and challenges accessing services and resources [8,9]. Inequities are particularly pronounced among those with multiple marginal intersectional identities (e.g., individuals who are both autistic and Black, Indigenous, and People of Color [BIPOC]) [10]. Renewed attention to these disparities emerged during the COVID-19 pandemic because ND individuals experienced more severe illness, greater risk of hospitalization, and almost twice the case fatality rates of the general population [1,2]. They also experienced significant mental, social, and financial strain as well as heightened health and safety concerns [11].

## 3 Inclusive public health communication

During the earliest days of the COVID-19 pandemic and up until the present, there is an opportunity to address health inequities for ND populations utilizing *inclusive public health communication*, defined as health-related messaging that includes and prioritizes ND populations in public health preparedness and response efforts [3]. Research that emerged during the pandemic suggests that there are three key aspects of inclusive public health communication: 1) ensuring message accessibility (e.g., information is delivered via multiple modalities in plain language); 2) ensuring that public health recommendations promote connectedness and inclusion, rather than segregation and exclusion; and 3) ensuring that public health communicators are trained to support the diverse needs of marginalized populations [3].

## 4 Barriers to inclusive health communication

The literature underscores several extant barriers to effective inclusive public health communication efforts for ND individuals [1,2]. First, public health communication efforts for ND populations—and all marginalized populations, for that matter—have inadequately aligned with intersectionality, defined as a theoretical framework for understanding how multiple social identities intersect and create health disparities [12]. Indeed, there is a long history in public health of conceptualizing marginalized groups as monolithic, rather than recognizing the myriad ways in which multiple social identities interact and compound to affect lifelong health [12]. To be sure, the concurrent social movements of the COVID-19 pandemic—including Black Lives Matter—spurred a renewed recognition of the importance of intersectionality in understanding and addressing health disparities through public health efforts [13]. Public health communicators may be challenged to distinguish between intersecting identities, social positions, processes, and policies or other structural factors [14], and struggle to deliver public health messages attuned to the diversity within particular groups.

An additional barrier to inclusive public health communication is implicit and explicit bias historically interwoven in public health and healthcare [15]. Bias refers to unconscious ideas about certain people or groups, operating from outside of the person's awareness (implicit) as well as conscious ideas and beliefs that often result in negative or exclusionary behaviors towards certain people or groups (explicit) [16]. Bias often originates from lack of education and training on the part of the health communicator [17].

The COVID-19 pandemic underscored the ways in which public health messages have excluded the ND population. A New Yorker article, for example, featured several hearing- and vision-impaired adults who expressed concerns about the inherent biases they face in receiving equitable treatment [18]. This article described multiple examples of misalignment between the ND population's needs and experiences and public health messages, including: “Many deaf-blind people … were cut off from basic information. The daily White House briefings provide no sign-language translations. Data and statistics about the pandemic, its spread, and its numbers are presented in a visual format in the media.” [18].

Bias leads to poor health via multiple mechanisms. As indicated above, it can indirectly prevent people from accessing services or receiving health-promoting resources [19]. Experiencing bias can also directly induce heightened physiological stress responses that lead to increased cortisol and accumulate to create physical “wear and tear” or allostatic load. Other stress responses include maladaptive coping behaviors (e.g., substance use and disordered eating) and heightened vulnerability to emotional and interpersonal challenges [19].

## 5 Facilitators to inclusive health communication

There are several factors that can potentially counteract these barriers and facilitate effective inclusive public health communication efforts. Social media, for example, can serve as a tool for disseminating health-promotion interventions and resources; addressing health access and literacy issues; and expanding research and evaluation efforts [20]. Indeed, during the COVID-19 pandemic, social media was a widely utilized public health tool and ND communities were particularly active on social media during the pandemic [21]. At the same time, there remain multiple roadblocks to ND populations’ utilization of social media for health purposes, including lack of accessibility, threats of victimization and discrimination, and likelihood of misunderstandings and distress [22].

In effect, for social media—or any strategy, for that matter—to be effective, establishing trust and reliability is a critical pre-requesite [23]. Trust between ND populations and healthcare institutions, however, has been degraded by a long-standing history of misinformation in research and practice spheres, particularly among those with intersectional marginalized identities [24]. This has been fueled by exclusion or exploitation of ND individuals in research as well as misinformation about vaccines that has permeated autistic and other ND populations for decades [25].

The literature suggests several key strategies for establishing trust with ND communities. First, there is a general need to increase communication with the ND community, which includes fostering authentic connections, maintaining an open dialogue, and promoting participatory approaches in health-related efforts [26]. In practice, participatory approaches may entail healthcare or service providers holding listening sessions or focus groups; engaging community groups working on the ground with ND populations; or conducting individual- or family-level outreach [26]. Communication efforts must be delivered with accessibility at the forefront [27]. Indeed, there are ongoing technical assistance efforts that teach public health stakeholders about accessibility by providing training regarding utilizing multiple communication modalities, flexible dissemination strategies, and other tailored approaches in virtual and in-person public health communication efforts [27].

Increasing communication and relationship building is one of the first steps towards establishing trust; efforts should also engage ND populations and their families and caregivers as experts [26]. In practice, this means that communication strategies should not only seek to forge close relationships, but also foster *collaborative relationships* wherein the balance of power is equal between professionals and ND populations [26]. Existing efforts are underway involving partnerships with ND populations in the creation of healthcare-related materials [28]. There is a need to continue and expand these efforts, by, for example, co-creating public health materials or iteratively refining strategies based on continued feedback during and post-COVID-19. Across all of these efforts, communication must promote acceptance and support of differences [5]. This entails acknowledging the vast heterogeneity in ND populations and embracing different preferences, processing styles, and priorities within these diverse populations. These differences should be recognized as such, rather than as deficits or problems to be “fixed.” [29].

Finally, ensuring public health communication is credible is an important facilitator of inclusive public health communication. This involves ensuring that approaches are not only science-based, but also delivered by trustworthy messengers—including scientists and individuals with lived experience—in a manner that is clear, direct, and non-ambiguous [30]. Given rampant scientific mistrust within ND communities [25], credible messaging is necessary for re-establishing trust and confidence. This is particularly important in light of the lasting effects of inaccurate vaccine messages towards autistic and other marginalized communities, for example [25].

## 6 Research, practice, and policy recommendations

Taken together, the available evidence reviewed in this commentary on inclusive public health communication suggests several areas for future research. First, there is a need to further understand the barriers to inclusive public health communication. There are limited studies that seek to understand how ND stakeholders understand and conceptualize intersectionality and integrate it in their health communication efforts. This information would be helpful in planning efforts to develop or refine public health communication. Second, despite the well-established existence and consequences of bias, there are limited research studies that seek to understand how ND stakeholders—including those who work in healthcare or direct service provision, governmental or academic positions, and others—understand and address bias in their communication efforts. This lack of information stymies efforts to reach ND populations—who anticipate and experience bias in their interactions with the healthcare system [5]—through public health communication efforts. Third, there is a need for research on the facilitators of inclusive public health communication. As an example, there remains a paucity of research on social media utilization of ND stakeholders and the types of reforms necessary to achieve inclusive public health communication for ND populations. Finally, there is a need for research to identify strategies for establishing and maintaining trust with the ND community in public health efforts. Importantly, all research efforts should be developed in collaboration with key ND stakeholders.

In addition to addressing key research areas related to inclusive public health communication, it is also important to prepare the current workforce—including direct service providers, physicians, policymakers and other ND stakeholders—to be effective inclusive public health communicators. In developing and pilot-testing efforts to improve inclusive public health capacities, learning objectives should directly address impediments to inclusive public health communication, including intersectionality and healthcare bias. Trainings and educational approaches should leverage multiple social media platforms and support participants in differentially utilizing these platforms to engage targeted audiences, including researchers, practitioners, policymakers, and the media. Content should be periodically re-assessed to ensure it is responsive to the dynamic nature of public health priorities. It may be ideal to offer trainings virtually so as to reach a broad, diverse audience, comprised of different ND stakeholders. Pedagogy may be most effective if geared towards application and translation of existing knowledge and values, rather than towards utilizing didactics and promoting content knowledge. This includes emphasizing how concepts such as intersectionality directly translate to real-world health implications. Social media skills should be fostered in the context of public health and health equity efforts, rather than through a more generalized approach disconnected from these efforts.

## 7 Conclusion

Inclusive public health communication represents a promising approach to addressing health inequities for the ND population. Inclusive public health communication requires the commitment of a range of ND stakeholders, including healthcare and service providers, families and caregivers, and ND individuals themselves. Currently, however, lack of integration of intersectionality into public health efforts—as well as implicit and explicit bias—impede efforts to implement and scale effective inclusive public health communication strategies. There are opportunities to utilize tools such as social media to promote the health and well-being of ND individuals. Most importantly, it is critical for ND stakeholders to first establish trust with ND populations. Future research, quality improvement, and evaluation initiatives that seek to bolster inclusive public health capacities are necessary next steps towards this end.

## Declaration of competing interest

The authors declare that they have no known competing financial interests or personal relationships that could have appeared to influence the work reported in this paper.
